# Identifying dispersal events of red foxes (*Vulpes vulpes*) using early warning signals

**DOI:** 10.1186/s40462-025-00579-w

**Published:** 2025-07-29

**Authors:** Felicitas Oehler, Janosch Arnold, Klaus Hackländer, Johannes Signer, Stéphanie C. Schai-Braun, Robert Hagen

**Affiliations:** 1 Wildlife Research Unit, Agricultural Centre Baden-Württemberg, LAZBW, Aulendorf, Germany; 2 Institute of Wildlife Biology and Game Management, Department for Ecosystem Management, Climate and Biodiversity, BOKU University, Vienna, Austria; 3 Forest Research Institute Baden-Württemberg, Freiburg, Germany; 4 Deutsche Wildtier Stiftung (German Wildlife Foundation), Hamburg, Germany; 5 Wildlife Sciences, Faculty of Forest Science and Forest Ecology, University of Goettingen, Göttingen, Germany

**Keywords:** Dispersal, Regime shift, Early warning signal, Net squared displacement, Mechanistic range shift analysis

## Abstract

**Background:**

Many animals disperse to find their own territory, mates to reproduce or suitable environments to live. Dispersal can be described as a three-phase process consisting of two stationary phases (S_1_ and S_2_) at the beginning and the end of a dispersal event. These stationary phases are temporally separated by a transient phase (T), where the animal moves from S_1_ to a new area S_2_ in space. The net squared displacement (NSD) is a frequently used metric to identify these phases from animal tracking data.

**Methods:**

We tested whether early warning signals (EWSs) on time series of the NSD, can be used to predict dispersal events. To identify EWSs we conducted a rolling window approach and evaluated the dispersal events by performing a spatial cluster analysis with the mechanistic range shift analysis (MRSA). We used data from 22 GPS-collared red foxes (*Vulpes vulpes*) as an example of a mammal species in which the juvenile (sub-) adult transition usually involves dispersal.

**Results:**

Applying EWSs resulted in the identification of both transitions from S_1_ to T and from T to S_2_. For 10 individuals we detected EWSs. For 8 out of these 10 individuals (80%) we identified a spatial shift between S_1_ and S_2_ via a MRSA. Accordingly, for 8 out of 22 individuals (36%) we observed a transient phase (T) which led to a major and persistent transformation of red fox locations.

**Conclusion:**

Even though the identification of dispersal events based on movement data is challenging using well known techniques such as state space models or the MRSA, our results suggested that EWS in combination with MRSA is appropriate to detect and identify dispersal events in radio-collared mammals. Thus, in the context of identifying dispersal events using EWSs we recommend to evaluate the existence of stationary and transient phases using the MSRA. The benefit of using EWSs is the calculation of the NSD and simple statistics (standard deviation, autocorrelation) and no requirement of high resolution tracking data. Additionally, transitions to the stationary or transient phase might be detected where home range calculations are not possible.

## Introduction

Dispersal is a key process in ecology, influencing demography and evolution of populations [1]. It is essential for many animals to find their own territory, to find mates or suitable environments to live [2]. Thus, dispersal is crucial to increase fitness through higher reproductive success by avoiding kin competition or inbreeding [3–5] or by leaving areas where habitat is degraded due to climate change [6], urbanization [7] or fragmentation [2]. Thus, dispersal is often triggered by resource and inter- and intraspecific competition [2] and is varying between the prevalent social system [8]. Furthermore, dispersal can be linked to sexual maturation [2, 9, 10] occurring during the transition from juvenile to (sub-) adulthood [2, 11]. Dispersing individuals influence the environment. They can spread plant seeds or diseases, introduce new predator-prey conflicts or alter habitat compositions [1].

One species relevant to study its dispersal behaviour, is the red fox (*Vulpes vulpes*). Red foxes disperse mainly as subadults during autumn [12] and they have the potential to spread diseases [13] and influence other species by predation or competition [14, 15]. The dispersal length of red foxes vary between distances smaller than five kilometres to long distances up to a few hundred kilometres [16, 17]. The dispersal distances depend on environmental conditions, for example on food or shelter availabilities, intra- and interspecific competitions. Dispersal duration shows high variability between one day and multiple weeks [17]. The space use patterns of red foxes can be complex, which makes it challenging to classify individuals as dispersers. While some dispersal movements are easy to identify visually based on GPS-points, other dispersal movements might not be as clearly detectable, because the dispersal distance is small or individuals shift their home ranges with time for which home range definitions are not easy to conduct [18, 19]. Thus, limitations in methodology and study design often introduce difficulties to clearly classify dispersal events [17, 19, 20]. Therefore, we introduce a simple method identifying dispersal events in movement data of red foxes, by looking at spatial and temporal movement characteristics. We use the red fox (*Vulpes vulpes*) as study species as red foxes show a variety of movement patterns during their life-history [21], among them dispersal movements [22, 23].

In the context of movement ecology, dispersal in mammals can be described as a three phase process consisting of two spatially distinguished stationary phases (S_1_ and S_2_), temporally separated by a transient phase (T) [24, 25]. Movements in stationary phases are spatially stable, reflecting e.g. daily foraging movements in a home range [26–29]. Movement patterns will likely differ during the transient phase compared to stationary phases or to patterns during extraterritorial movements during breeding or mating time [2, 29–31]. Individuals move often fast and directed between two patches during T to save energy [29, 32–34] and to minimize risk [35]. The net squared displacement (NSD) is frequently applied to identify dispersal and to describe this three phase process in free ranging individuals [25, 36, 37]. The NSD is the squared value of the Euclidian distance from the start location to each subsequent location. When an individual begins to disperse, the change in space and in the movement pattern might be observed by linking the movement data to methods, which detect changes in movement patterns. Multiple methods for detecting changes in movement exist such as time series analysis, quantitative descriptions of underlying movement patterns or analysis of state space models [38, 39]. One example is the classification of movement data into broad movement strategies (migration, dispersal, nomadism, sedentarism) using NSD and latent state modelling [40]. Movement strategies can be analysed as well via the mechanistic range shift analysis (MRSA), using estimations of spatial and temporal range shift parameters as e.g. areas of ranges, centroids and durations of transitions [41]. However, identifying demographic processes such as migration or dispersal based on movement data including approaches based on the NSD remains very challenging [40–42]. Difficulties to classify dispersal are introduced due to the complexity and variability of movement data [40], which makes it nearly impossible that a single method identifies correctly all possible segmentation parts in a movement path and thus reliably identifies dispersal events [38]. Therefore, a combination of at least two methods might be useful, e.g. first, the analysis of movement parameters (NSD) and second, the analysis of spatial measures (MRSA), as recommended by Cagnacci et al. [43].

In addition, movement ecology might benefit from the application of concepts and methods of dynamical systems including the regime shift concept to identify dispersal in collared individuals. The regime shift concept describes the transition process of a dynamical system from one state (regime) to another (State 1 to State 2, in our example S_1_ to S_2_). The phase of transition is usually characterised by a drastic change [44]. Early warning signals (EWSs) can be applied to a wide range of real-world systems to detect this drastic change [44–47]. Aside from other metrics, the application of the standard deviation (sd) and the autocorrelation function (acf) are frequently used to detect an EWS [46–48]. In the context of movement ecology and dispersal, such a transition might be linked to an increase in temporal autocorrelation of the NSD for low lags and by an increase in the variance of the NSD. For S_1_ and S_2_ it is already known that the NSD fluctuates around a specific value [25]. When EWS indicated a transition between states, the MRSA can be used to prove whether an actual shift in the spatial distribution happened and thus, as an evaluation of dispersal. In this study, we introduce a novel approach identifying dispersal movements in mammals using a combination of (a) EWSs based on statistics of the NSD and (b) MRSA based on GPS-points.

## Materials and methods

The study was conducted in a rural area (142,400 ha) in South Germany (47°51’18.0"N 9°32’11.8"E), representing a heterogeneous landscape. We equipped 26 red foxes with Global Positioning System (GPS) collars (‘Collar 1C’; 170 g, e-obs, Grünwald, Germany and ‘Tellus ultra light’, 213 g, Followit, Lindesberg, Sweden) between October 2020 and March 2023. All procedures were approved and accepted by the ‘Regierungspräsidium Tübingen’ under the animal protection law, registered as LAZ4/19G and supervised by veterinarians. Detailed description about the landscape and the handling procedure of red foxes (trapping and collaring) are described in the study by Oehler et al. [29]. GPS collars collected a GPS location every 10 min if the collard animal was active and every 1 h if the collard animal was inactive (e-obs collars) or every 20 min during night and every 3 h during day (Followit collars).

To identify movements, we first removed spatial outliers (for detailed information see Oehler et al. [29]) and excluded all GPS-signals taken within the first 24 h to exclude trapping bias [49]. Four individuals that collected less than one month GPS data were excluded from further analyses, which resulted in 22 individuals. Duration of data collection per individual is summarized in the study by Oehler et al. [29]. Following Singh et al. [36] on the classification success of movement patterns using the NSD, we subsampled our dataset to a sampling rate of 12 h with a 1 h tolerance. Singh et al. [36] showed that high-resolution data, reflecting 6 to 48 locations per day, did not improve the classification success of movement patterns using NSD, emphasizing that 1 location per day is sufficient. For our red fox data more than 98% of all subsequent locations of any collared individual lied within a period of 12 h (± 1 h). Thus, the assumption about a continuous time series for further analysis of the NSD is met. We calculated the NSD as the squared Euclidian distance from the start location, the trapping location plus one day of data collection, (x_s_,y_s_) to each subsequent location (x_t_,y_t_) (Eq. 1):

1 $$NS{D_{s,t}} = {({x_t} - {x_s})^2} + {({y_t} - {y_s})^2}$$

We transformed the NSD to nNSD, where the NSD values are transformed to kilometers (Eq. 2):

2 $$\:nNSD\:\left[km\right]\hspace{0.17em}=\hspace{0.17em}SQRT\left(NSD\right)/1000$$

We then calculated the standard deviation (sd) and the temporal autocorrelation function (acf) of the nNSD time series. The sd can be related to the variance: $\:sd=\frac{1}{n-1}*\sum\:_{t=1}^{n}{({nNSD}_{t}-\mu\:)}^{2}$, where µ is the mean of the nNSD time series. The acf with lag length 1 of the nNSD is: $\:acf=\frac{E\left[\left({nNSD}_{t}-\mu\:\right)\left({nNSD}_{t+1}-\mu\:\right)\right]}{{\sigma\:}_{z}^{2}}$, where E is the expected value, µ is the mean nNSD and σ = variance of the nNSD_t_. On basis of the sd and the acf we calculated the signal strength as:

3 $$\:Signal\:strength\:=\left|\:f\right(sd\:\left(nNSD\right)\left)\right|w\:+\:\left|\:f\right(acf\:\left(nNSD\right)\left)\right|w$$

where *f(x)* is the function of the standardised time series of x (mean = 0, standard deviation = 1) for a specific temporal window *w*, using the rolling window approach [46] via the R-package earlywarnings [50]. Second we applied a k-means cluster algorithm provided via the R-package marcher [41, 51] to estimate two distinct spatial clusters corresponding to S_1_ and S_2_ together with an estimated time (t_1_) at which the transition started.

### Rolling window and the existence of two spatial distinct clusters

Each rolling window contained data of red fox movement of 5 days. Thus, for a sampling rate of 12 h we used 10 GPS points to calculate estimates for sd and acf of the nNSD. The case that the signal strength (Eq. 3) exceeded a threshold of 3.92, the sum of 2 σ (sd) and 2 σ (acf), was used as an indicator for a warning signal. Thus, a warning signal is given in all situations for which the classical 2 σ method [46, 47] pointed to a warning signal. Even though the 2 σ method is a robust classification method to identify changes dependent on an unweighted sum of two statistics such as the sd and the acf [47], we decided to use a more general approach based on the sum of the absolute values of both statistics (Eq. 3). This is important for analysing the NSD since a dispersal event might be frequently accompanied by a decrease of the acf of the NSD, whereas the sd of the NSD increases. Thus, our EWS accounted for situations where the direction of the two signals were different. Furthermore, we conducted a sensitivity analysis to investigate the impact of increasing or decreasing the threshold by 10%. For more details, see Appendix ‘Sensitivity Analysis’.

If the signal strength (Eq. 3) exceeded the threshold of 3.92 for a certain number of subsequent windows, we interpreted this persistence as an early warning signal (EWS). Clements et al. [52] showed the benefit of interpreting a set of subsequent warning signals as an EWS to reduce the false positive rate. Southall et al. [47] demonstrated that the optimal number of consecutive warning signals is likely higher than two to initiate a robust EWS. Thus, we investigated whether the classification ‘dispersal event’ depended on the number of subsequent warning signals applied, interpreting either three (36 h), four (48 h) or five (60 h) subsequent warning signals as an EWS.

For individuals where we detected a shift between S_1_ and T, we further tried to identify the transition between T and S_2_. Therefore, we used the same rolling window method for the reversed time series of the nNSD. Only for those individuals where we received an EWS for both, the transitions S_1_ to T and T to S_2_, we evaluated the findings by the existence or absence of two spatial distinct clusters, using a k-means cluster algorithm provided via the R-package marcher [41, 51]. We used the nonlinear least squared model available via ‘estimate_shift()’ and ‘test_rangeshift()’ of the R-package marcher on the movement track. The former can be used to identify 2 spatial clusters corresponding to a 2-dimensional Kernel estimator for the home ranges for S_1_ and S_2_. Subsequently, the latter performs a test whether a shift happened between S_1_ and S_2_. To finally classify an individual as disperser, we further used two criteria. First, that the distance of the centroids of the home ranges for S_1_ and S_2_ had to be at least 2 km (species specific distance), and second that the 50% estimated area of use did not overlap but contained at least two GPS fixes. For red fox the assumption about the distance between the centroids of S_1_ and S_2_ of more than 2 km reflects the circumstance that home range of red foxes within heterogeneous patches of multiple agricultural fields is known to be rather small [53]. In this context a 2 dimensional Kernel estimator with a radius of 2 km is associated to a home range size of about 13 km^2^, which is in line with other studies on home range size of red foxes in rural, suburban areas [54–56]. All data analysis was conducted with R version 4.1.3 [57].

## Results

We collected 247,011 GPS points. After subsampling to locations every 12 h 4,489 GPS points remained for our analysis.

When looking at two spatial clusters in movement data of the 22 individuals without additional criteria the MRSA identified for 19 individuals not only spatial clusters but also a transition time (Table 1). For the condition ‘no overlap of 50% (95%) kernel estimator’ the number of individuals for which the stationary phases could be linked to a spatial cluster was 14 (4) out of 22 individuals (Table 1).

For a sampling rate of 12 h and a window size of 10 subsequent locations, we detected potential transitions S_1_ to T and T to S_2_ for 11, 10 and 7 individuals out of 22 individuals (Tables 1 and 2). The detection was dependent on the number of subsequent warning signals for 3, 4 or 5 subsequent rolling windows exceeding the threshold of 3.92, respectively (Table 2).

For those individuals we calculated the distance of the centroids of the kernel estimators. We found that a threshold value of 2 km lead to a number of 8 out of 10 (11) or 5 out of 7 individuals (Table 2) for which the EWS was truly associated to a spatial transition. Using a threshold lower than 2 km, such as 1 km led to the marking of individuals as disperser that did not disperse (for example FM11 using the EWS or FM11, FM12, FM15 using the MRSA, see Table 1).

**Table 1:** Classification of individuals into dispersers (IDs in bold). A warning signal was achieved when the signal strength (Eq. 3) exceeded the threshold of 3.92 for 5 (dark gray)/ 4 (gray)/ 3 (light gray) subsequent rolling windows. If no distinct clusters were identified, marcher either could not detect any cluster, an estimated cluster contained only one point or even the 50% estimated area of use overlapped. The timing of the EWS is the time where the third, fourth or fifth window exceeded the threshold. The time in the brackets shows the duration of the window with start and end time of the window. IDs with the abbreviation FM are males and FF are females

	Rolling window detection of warning signals S_1_ to T			Rolling window reverse detection of warning signals T to S_2_			Marcher			Detected timing of transition S_1_ to T		
ID	Threshold for subsequent EWS signals			Threshold for subsequent EWS signals			2 distinct clusters identified		Distance between centroids [km]	quickfit()	estimate_shift()	EWS (4 signals) [window]
	5	4	3	5	4	3	yes					
							no overlap of 95% kernel estimator	no overlap of 50% kernel estimator				
**FM1**	✓	✓	✓	✓	✓	✓	✓	✓	16.21	7.5	1	4 [1–10]
FM2	-	-	✓	-	-	-	-	-	-	3	5.98	-
**FM4**	-	✓	✓	-	✓	✓	-	✓	9.28	98.5	101.11	99 [96–105]
**FM5**	✓	✓	✓	-	✓	✓	✓	✓	10.46	53.5	56	51 [48–57]
**FM6**	✓	✓	✓	✓	✓	✓	-	✓	6.65	6.5	9.55	5 [2–11]
FM7	-	-	-	-	-	-	-	-	0.53	-	-	-
**FM9**	✓	✓	✓	✓	✓	✓	✓	✓	7.43	34.5	37.6	33 [30–39]
FM10	-	-	-	-	-	-	-	-	-	0.5	-	-
FM11	✓	✓	✓	✓	✓	✓	-	✓	1.52	8.5	15	7 [4–13]
FM12	-	-	-	-	-	-	-	✓	1.23	36.5	41	-
FM13	-	-	-	-	-	-	-	✓	0.62	154.5	-	-
FM14	-	-	-	-	-	-	-	-	0.56	10.5	-	-
FM15	-	-	-	-	-	-	-	✓	1.01	137.5	-	-
FM16	✓	✓	✓	✓	✓	✓	-	✓	0.48	84.5	84	89 [86–95]
**FM17**	-	✓	✓	✓	✓	✓	-	✓	4.41	0.5	51	50 [47–56]
FM19	-	-	-	-	-	-	-	-	0.14	18.5	-	-
**FF1**	✓	✓	✓	✓	✓	✓	-	✓	2.46	11.5	36.8	11 [8–17]
FF3	-	-	-	-	-	-	-	✓	0.77	183.5	160	-
**FF4**	✓	✓	✓	✓	✓	✓	✓	✓	3.08	120.5	123.4	100 [97–106]
FF5	-	-	✓	-	-	✓	-	-	0.44	-	-	-
FF6	-	-	-	-	-	-	-	-	0.44	-	-	-
FF8	-	-	-	-	-	-	-	-	0.25	0	-	-

For our data we interpreted 4 subsequent warning signals as an EWS which identified both the transition from S_1_ to T and from T to S_2_ (Table 2). The evaluation of these transitions using the MRSA showed that 80% of the detected transitions were associated to a spatial shift (Table 2).

**Fig. 1:**
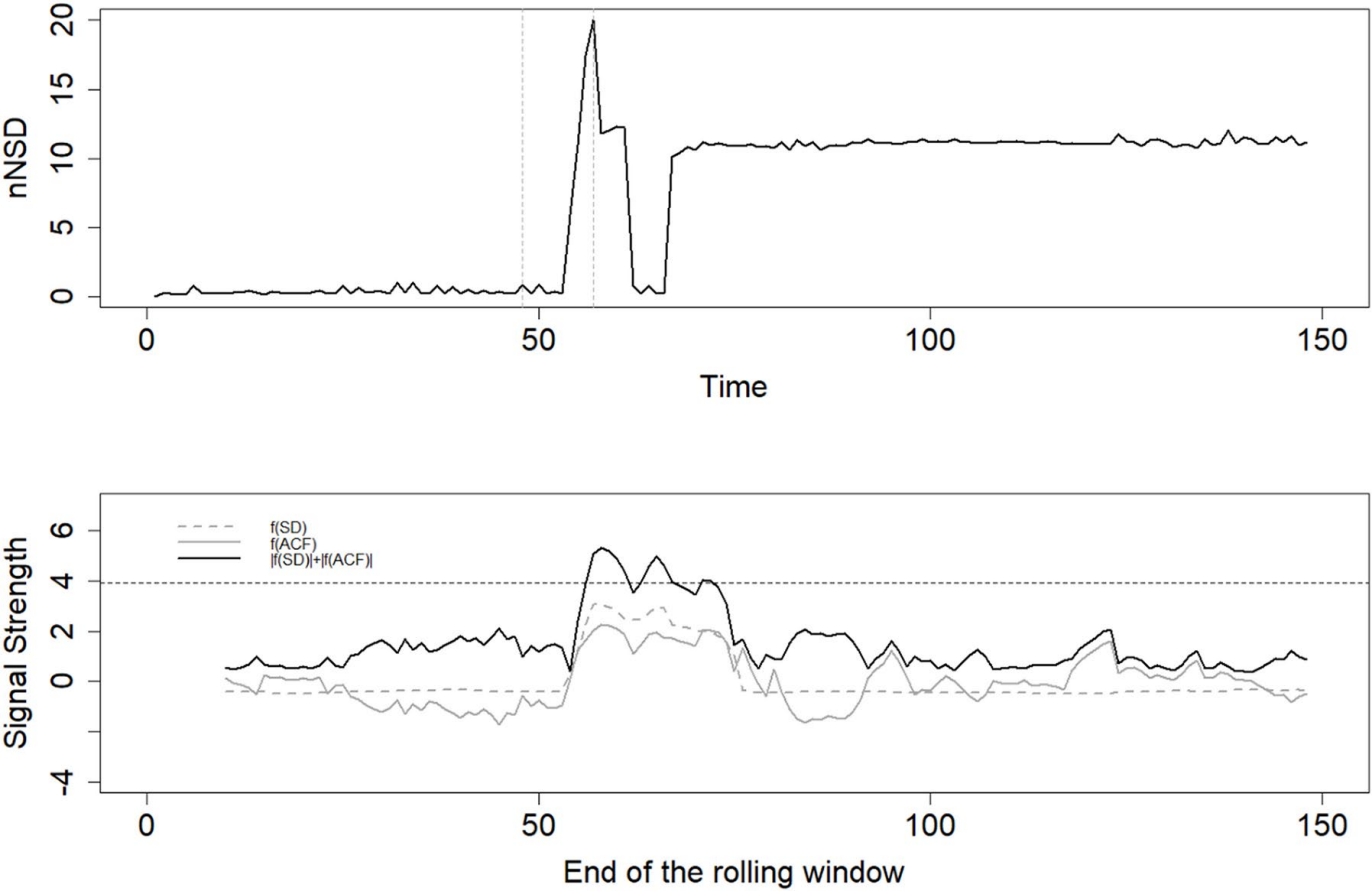
The distance from each GPS point to the start location (nNSD, top panel) of the red fox individual FM5 including the beginning of the rolling window in which the fourth EWS was detected (vertical dashed line on the left) and the end of the rolling window (vertical dashed line on the right). The bottom panel shows the signal strength, the sum of the absolute values of f(sd) and f(acf) of the nNSD (Eq. 3, black solid line) and f(sd) (dashed line) and f(acf) of the nNSD (gray line). The threshold of 2*standard deviation (f(acf)) + 2*standard deviation (f(sd)) is visualized as dark gray dashed line

**Fig. 2:**
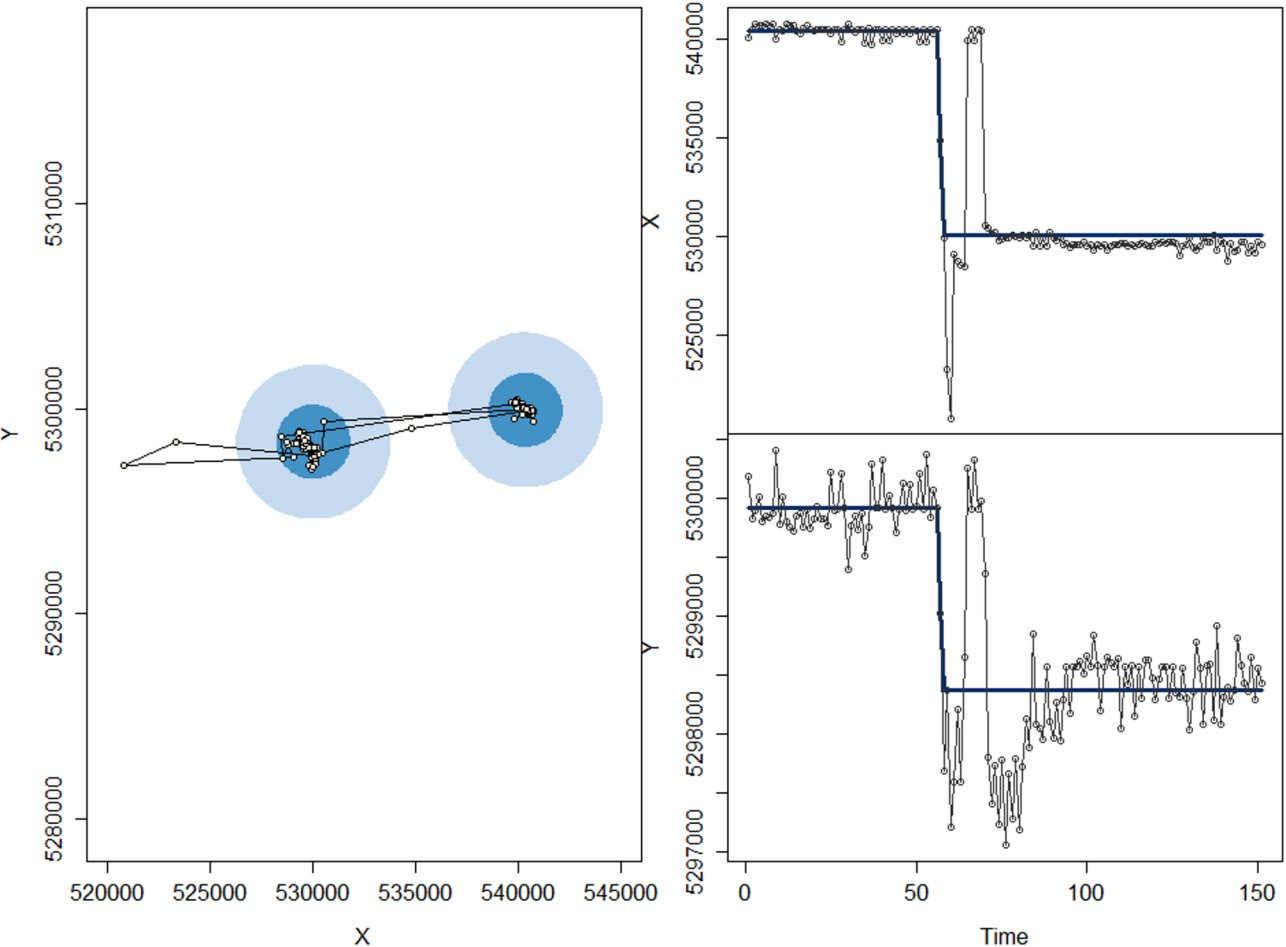
Relocations with estimated 2-dimensional home ranges for individual FM5 (left panel). The dark blue area shows the 50% estimated area of use and the light blue area the 95% estimated area of use. Time series of the the x and y coordinates (right panels). The plot was generated using the R-package marcher [51]

When using EWSs in combination with MRSA, 8 out of 22 red foxes were classified as disperser (2 out of 6 females, 6 out of 16 males, Table 1). For those individuals, the identified transient phase (T) led to a major and persistent transformation of red fox locations (Figs. 1 and 2 for an example, or compare Fig. 5 of the study of Oehler et al. [29]). Dispersal distance varied between 2.46 km and 16.21 km (Table 1), showing the distance between the two centroids of S_1_ and S_2_. The duration of the transient phase varied between 1 and 28 days.

In sum our results show that individuals can be classified as dispersers with the combination of EWSs and MRSA if:

i. the rolling window method detects EWSs for both transitions (S_1_ to T & T to S_2_) for four subsequent signals and
ii. the MRSA identifies two distinct clusters (without overlap of the 50% estimated area of use), where the distance between the two cluster centroids is > 2 km.

**Table 2:** Left side shows the number of individuals for which the signal strength (Eq. 3) exceeded the threshold of 3.92 for 3, 4, 5 subsequent rolling windows. The EWS detection was evaluated by the detection of spatial distinct clusters. The right side shows the number of individuals for which two spatial distinct clusters were identified without receiving EWSs. Distinct spatial clusters means that our conditions are met: the centroids had an Euclidian distance of more than 2 km while at least the 50% kernel estimates for the home ranges did not overlap

	Number of individuals with EWS (S_1_ to T and T to S_2_)	Number of individuals for which 2 distinct spatial clusters were detected	Number of individuals without EWS	Number of individuals for which 2 distinct spatial clusters were detected
3 subsequent rolling windows	11	8 of 11 (72.7%)	11	0 of 11 (0%)
4 subsequent rolling windows	10	8 of 10 (80%)	12	0 of 12 (0%)
5 subsequent rolling windows	7	5 of 7 (71.4%)	15	3 of 15 (20%)

## Discussion

In this study, we were able to detect dispersal movements of red fox individuals using the standard deviation and the autocorrelation function of the NSD. We evaluated these dispersal events by the existence of two spatial distinct clusters (S_1_, S_2_) via a MRSA. Based on the findings of our study the application of EWSs is appropriate in detecting dispersal events (Table 1). Using MRSA alone resulted in either an overestimation or underestimation of the number of dispersers (Table 1). Thus, our results highlight the limitation of identifying dispersal events by using MRSA alone. However, evaluating the transitions identified by the EWS using MRSA with an additional criteria about the distance of the home range centroids led to qualitative good and robust results. Using a combination of at least two methods to correctly classify dispersal events was already suggested by Cagnacci et al. [43].

The performance of detecting the shifts between S_1_ and T and T and S_2_ depended on the applied threshold criteria for the number of subsequent warning signals interpreted as EWS (Table 2). Southall et al. [47] demonstrated already that the optimal number of consecutive warning signals is likely higher than two to initiate a robust EWS. Due to the highest classification success with 4 subsequent warning signals, we interpreted 4 subsequent warning signals as an appropriate EWS to identify transitions in red fox movement patterns. However, the number of subsequent warning signals interpreted as an EWS will likely depend on sampling rate (see Appendix for results of the classification success using a sampling rate of 6 h (± 1 h) instead of using a sampling rate of 12 h (± 1 h)). Using a sampling rate of 6 h, and thus more frequent spatial data suggests that the application of EWSs is suitable in detecting the transient phase for individuals where home range calculations are not possible. Home range calculations might be difficult to conduct for individuals where data collection started or stopped during transient phase, where data points are missing to conduct home range analysis or, where home ranges shift with time and no clear structure exists. For future applications EWSs can be applied while collecting data. This real-time analysis could improve the data collection by changing the collection frequency to better understand the movement ecology during dispersal or just to save battery and to be able to collect data according to the research question. Thereby, lower data resolution can be used to identify dispersal, as presented in this study, but higher resolution data is needed to analyse movement and behavioural patterns in time and space, as conducted in the study by Oehler et al. [29].

The rather small dispersal distances between 2.46 km and 16.21 km detected in our study reflect dispersal distances in rural heterogeneous landscapes, where landscape productivity, competition, resource availability and population density influence dispersal distances [15, 16]. This shows, that food availability and fox density is high in our study area, compared to Scandinavia for example, where more long distance dispersals were observed [16].

By identifying the shift from stationary to transient and back to stationary phases, our results support the concept of dispersal as a three phase process (S_1_, T, S_2_, cf [25]). The successful identification of a dispersal event using the rather simple theoretical concept of early warning signals suggest that dispersal can be understood as an example of a regime shift. Actually, the regime shift concept was applied to many different contexts (biology, physics, medicine, sociology – cf [44, 58]). and different levels of hierarchy (from low to high: an individual, a population, an ecosystem, the earth). In this context it was shown that a set of early warning signals, including the sd and the acf have the potential to indicate such a regime shift in any of these dynamical systems [44–47, 58–60]. Transferring this concept to dispersal, T reflects the shift and S_1_ and S_2_ reflect the regimes.

Since EWSs are appropriate to point to a drastic change in movement patterns of an individual independent of the species, this method has the potential to be applied to other species as well. However, EWSs applied alone neither provide an ecological background nor explanation for the observed behaviour. Thus, whether the change in movement is linked to dispersal or to any other drastic change such as migration or flight can only be answered by applying other analytical tools, as in our study by the MRSA. Linking ecological background to the applied method (EWS) proves the identification of dispersal, as shown by season in the study by Oehler et al. [29]. Dispersal predominantly occurs during autumn and winter [30]. The results of Oehler et al. [29] show that season is an important variable for the classification of dispersal phases of red foxes, where dispersal occurs during autumn and winter. Since Oehler et al. [29] used the definition of dispersal derived via the EWS and MRSA, as presented in this study, the framing of the findings by the ecology of the species proves the reasonable application of the EWS.

Our results about dispersal in red foxes and the transient phase suggested, that T, the transient phase might be split up in three phases for some individuals (for example the individual FM5 shown in Fig. 1). First a period characterised by an increase of the NSD-value, a phase where individuals explore areas including the later home range area, followed by a period rather characterised by a decrease of the NSD-value, reflecting the phase of coming back home. The third period is characterised by an increase of the NSD-value, reflecting the phase of the final shift. If this assumptions holds for the transient phase of other dynamical systems it might be of general importance for describing and identifying the transition and thus, for a better understanding of regime shifts. With the start and end date of the detected transient phase, the movement data of mammals can be segmented into stationary (S_1_ and S_2_) and transient movement phases which allows movement analysis specific to transient, exploratory or resident movement phases. Thus, movement patterns in these movement phases can be characterized and linked to habitat features for example to better understand movement patterns in specific movement phases as conducted by Oehler et al. [29]. We can further analyze along which structures dispersing individuals orient themselves to improve wildlife corridor planning or control disease spreads. Thus, this improved knowledge can help to mitigate human-wildlife conflicts.

## Conclusion

Even though the identification of dispersal events based on movement data is challenging using well known techniques, red fox’s dispersal events have been successfully identified with the application of EWSs in combination with MRSA in this study. Thus, we suggest that dispersal can be understood as an example of a regime shift and that EWSs in combination with MRSA are appropriate to detect and identify dispersal events in radio-collared vertebrates. The benefits of using EWSs to identify dispersal are (i) efficient calculation of simple statistics such as the standard deviation and the autocorrelation function (ii) no need for high resolution data and (iii) transitions to stationary or transient phase might be detected were home range calculations (MRSA) are not possible.

## Appendix

### Comparison of dispersal identification using a sampling rate of 12 h and 6 h

For our analysis we decided to interpret four subsequent warnings as an EWS. However, the appropriate number of subsequent warning signals might depend on the sampling rate. Here we used a sampling rate of 6 h (+-1 h) and compared the performance in identifying dispersal to our analysis presented in the main document using a sampling rate of 12 h (+-1 h).

For a sampling rate of 6 h we interpreted 8 subsequent warning signals as an EWS, to keep the time duration for constant warning signals equal to 48 h. Moreover, we further tested the performance of detecting dispersal for a sampling rate of 6 h still interpreting 4 subsequent warning signals as an EWS. Our results highlighted that a higher sampling frequency (6 h vs. 12 h) did not lead to a better performance in estimating dispersal (Table 4).

With a sampling period of 6 h.

- the number of individuals for which an EWS was detected and two distinct spatial clusters were identified was much lower (41.2% with 4 subsequent warning signals, 50% with 8 subsequent warning signals) than using a sampling frequency of 12 h (all values bigger than 71.4%).
- the EWS method was not able to detect all spatial transitions which were identified for a sampling period of 12 h.

We conclude for our data, that a sampling rate of 12 h should be preferred over a sampling rate of 6 h (Tables 3 and 4).

**Table 3:** Classification of individuals into dispersers (IDs in bold). A warning signal was achieved when the signal strength (Eq. 3) exceeded the threshold of 3.92 for 4 (6 h frequency, dark gray)/ 4 (12 h frequency, gray)/ 8 (6 h frequency, light gray) subsequent rolling windows. If no distinct clusters were identified, marcher either could not detect any cluster, an estimated cluster contained only one point or even the 50% estimated area of use overlapped. The timing of the EWS is the time where the fourth of 6 h frequency, the fourth of 12 h frequency or the eights of 6 h frequency window exceeded the threshold. The time in the brackets shows the duration of the window with start and end time of the window

	Rolling window detection of warning signals S_1_ to T			Detected timing of transition S_1_ to T			2 distinct clusters identified		Distance between centroids [km]
ID	Threshold for subsequent EWS signals			EWS (4 signals) [window] -6 h	EWS (4 signals) [window] -12 h	EWS (8 signals) [window] -6 h			
	4 (6 h)	4 (12 h)	8 (6 h)				no overlap of 95% kernel estimator	no overlap of 50% kernel estimator	
**FM1**	✓	✓	✓	4 [1–20] 24 h	4 [1–10] 48 h	8 [1–20] 48 h	✓	✓	16.21
FM2	✓	-	-	4 [1–20] 24 h	-	-	-	-	-
**FM4**	✓	✓	✓	169 [166–185] 1014 h	99 [96–105] 1188 h	173[166–185] 1038 h	-	✓	9.28
**FM5**	✓	✓	✓	95 [92–111] 570 h	51 [48–57] 612 h	99 [92–111] 594 h	✓	✓	10.46
**FM6**	✓	✓	✓	6 [3–22] 36 h	5 [2–11] 60 h	10 [3–22] 60 h	-	✓	6.65
FM7	✓	-	✓	193 [190–209] 1158 h	-	197 [190–209] 1182 h	-	-	0.53
**FM9**	✓	✓	✓	51 [48–67] 306 h	33 [30–39] 396 h	55 [48–67] 330 h	✓	✓	7.43
FM10	✓	-	-	4 [1–20] 24 h	-	-	-	-	-
FM11	✓	✓	✓	19 [16–35] 114 h	7 [4–13] 84 h	23[16–35] 138 h	-	✓	1.52
FM12	✓	-	✓	165 [162–181] 990	-	169 [162–181] 1014 h	-	✓	1.23
FM13	✓	-	✓	93 [ 90–109] 558 h	-	97[90–109] 582	-	✓	0.62
FM14	✓	-	✓	28 [25–44] 168 h	-	39 [32–51] 234 h	-	-	0.56
FM15	✓	-	-	168 [165–184] 966 h	-	-	-	✓	1.01
FM16	✓	✓	-	139 [136–155] 834 h	89 [86–95] 1068 h	-	-	✓	0.48
**FM17**	-	✓	-	-	50 [47–56]	-	-	✓	4.41
FM19	-	-	-	-	-	-	-	-	0.14
**FF1**	✓	✓	✓	4 [1–20] 24 h	11 [8–17] 132 h	8 [1–20] 48 h	-	✓	2.46
FF3	-	-	-	-	-	-	-	✓	0.77
**FF4**	✓	✓	-	233 [230–249] 1398 h	100 [97–106] 1200 h	-	✓	✓	3.08
FF5	✓	-	✓	207 [204–223] 1242 h	-	235 [228–247] 1410 h	-	-	0.44
FF6	✓	-	-	167 [164–183]	-	-	-	-	0.44
FF8	-	-	-	-	-	-	-	-	0.25

**Table 4:** Comparison of the results using a sampling period of 12 h and 6 h. Number of individuals which exceeded the threshold of 3.92 for 3, 4, 5 and 8 subsequent rolling windows. The EWS detection was evaluated by the detection of spatial distinct clusters

Sampling rate of 12 h	Number of individuals with EWS (S_1_ to T and T to S_2_) using a sampling rate of 12 h	Number of individuals for which 2 distinct spatial clusters were detected		Sampling rate of 6 h	Number of individuals with EWS (S_1_ to T and T to S_2_) using a sampling rate of 6 h	Number of individuals for which 2 distinct spatial clusters were detected	
		EWS detected	No EWS detected			EWS detected	No EWS detected
				4 subsequent rolling windows	17	7 of 17 (41.2%)	1 of 5 (20%)
3 subsequent rolling windows	11	8 of 11 (72.7%)	0 of 11 (0%)				
4 subsequent rolling windows	10	8 of 10 (80%)	0 of 12 (0%)	8 subsequent rolling windows	12	6 of 12 (50%)	2 of 10 (20%)
5 subsequent rolling windows	7	5 of 7 (71.4%)	3 of 15 (20%)				

### Sensitivity analysis

To investigate whether dispersal identification was affected by the choice of the threshold value (3.92) we conducted a sensitivity analysis. We calculated the sensitivity *S* to investigate whether the number of individuals identified as disperser *λ* depends on the setting of the threshold (u):

$$\:S\:=\:\left(\right|\lambda-\lambda_{i}|\:/\lambda)\:/\:\left(\right|u-u_{i}|\:/u)$$    

with *(|u-u*_*i*_*| /u) = 0.1* and *u* being the reference threshold value (3.92) while *u*_*i*_ being a parameter value which differs 10% from *u*.

A value of *S > 1* reflects that *λ* responds sensitive to variations in parameter *u*.

A reduction of the threshold by 10% led to an increase of the number of individuals detected by 10% (S = 1) while an increase of the threshold by 10% led to a reduction of the number of individuals detected by 30% (S = 3), Table 5. Thus, we assume that the method of the EWS to detect dispersal is rather unsuitable for threshold values exceeding 3.92 (2 Sigma + 2 Sigma).

**Table 5:** Individuals detected by our applied threshold (u) or by an increase (4.312) or decrease (3.528) of the threshold by 10%

ID\Threshold	3.528	3.92 (u)	4.312
FM1	X	X	X
FM2	-	-	-
**FM4**	**X**	**X**	**-**
**FM5**	**X**	**X**	**X**
**FM6**	**X**	**X**	**X**
FM7	-	-	-
**FM9**	**X**	**X**	**X**
FM10	-	-	-
FM11	X	X	X
FM12	-	-	-
FM13	-	-	-
FM14	-	-	-
FM15	-	-	-
FM16	X	X	-
**FM17**	**X**	**X**	**-**
FM19	-	-	-
**FF1**	**X**	**X**	**X**
FF3	-	-	-
**FF4**	**X**	**X**	**X**
FF5	x	-	-
FF6	-	-	-
FF8	-	-	-
EWS detected	11	10	7

## Data Availability

The datasets analysed during the current study are not publicly available due to further analyses of an ongoing PhD project.

## Abbreviations

GPS: Global Positioning System
NSD: net squared displacement
EWS: early warning signal
MRSA: mechanistic range shift analysis
